# Inferior and Impure Drugs

**Published:** 1872-09

**Authors:** 


### Editorial.

### Inferior and Impure Drugs.

Few physicians but are almost daily reminded of the great differences in the qualities of drugs, and yet we believe that few are sufficiently alive to the personal interest they have in the purity of the remedies they prescribe. The drug market is now made to suit the different grades of trade, and the name under which many articles are sold scarcely indicates anything of the nature of the drug. The reputation and success of a physician depends upon the qualities of the remedies dispensed almost as largely as upon his knowledge of disease, and many a patient leaves his medical adviser discouraged and unrelieved, who would have adhered to him with life-long confidence had his remedies been pure and reliable. In the cities, physicians are apt to leave this question of the purity of medicine with the druggist compounding their prescriptions. Alas! have left it without proper inquiry as to its purity and reliability.

In Buffalo we have as carefully selected medicines as the market affords, and we also have the lower and cheaper grades; that is, cheaper to the druggist, but never any cheaper, so far as we could learn, to the patient. The public know nothing of the value of medicines and are obliged to trust wholly to the fairness and judgment of the druggist or drug clerk. This being the case, the family physician is under every obligation to see that patients are treated fairly—that nothing unreasonable in price, and above all, nothing inferior in quality is sold to them, otherwise decoctions costing almost nothing will sometimes be furnished at the price usually charged for the same number of ounces of the most expensive solutions, and their prescriptions filled with inferior drugs. Our friends in the country may take warning, and with profit to themselves and patients EXAMINE, with greatest care, into the comparative and positive qualities of what they buy. If opium is bought as low in price as it can be, it is as poor as it can be. If Chloral is obtained below the market price of the very best, it is not the best chloral, and if Iod. of Potass. is now bought at former prices, it is not Iod. of Potass. But it is not the price of medicine, but its purity, and the duty of physicians in protecting communities from the competitions of trade, which we have mainly in view in these remarks. It is well for physicans not to interfere with the conveniences of patients in purchasing their medicines further than necessary to insure the very best of articles in the compounding of their prescriptions. This much is of untold importance to all parties—physician, patient and apothecay.

